# From disengagement coping to academic self-efficacy: the conditional role of emotional competence in STEM students

**DOI:** 10.3389/fpsyg.2026.1811939

**Published:** 2026-05-07

**Authors:** Aurelia Bulgac, Iulia Gonța, Ionuț-Răzvan Bratu

**Affiliations:** 1 Department of Psychology, The Faculty of Psychology and Educational Sciences, University of Bucharest, Bucharest, Romania; 2 Teacher Training and Social Sciences Department, University POLITEHNICA of Bucharest, Bucharest, Romania

**Keywords:** behavioral disengagement, coping, emotion regulation, emotional competence, self-efficacy, STEM education

## Abstract

**Introduction:**

STEM undergraduates often encounter high academic demands in contexts where stress may be normalized, potentially shaping coping strategies and academic self-efficacy. This study examined whether behavioral disengagement was associated with self-efficacy directly and indirectly via emotional competence, and whether psychological resources (hope, resilience, and optimism) moderated the association between disengagement and emotional competence.

**Methods:**

A sample of 131 first-year STEM students (M_age = 19.2, SD = 0.75) from a competitive academic context completed validated measures of coping, emotional competence, psychological capital, and self-efficacy. A moderated mediation model (Hayes Model 7) was tested using bootstrapping with 5,000 resamples.

**Results:**

The indirect association between behavioral disengagement and self-efficacy via emotional competence was significant. This indirect association was conditional: the negative association between disengagement and emotional competence, and the corresponding indirect association with self-efficacy, were stronger at average to high levels of hope, resilience, and optimism.

**Discussion:**

These findings suggest that emotional competence is meaningfully associated with the link between avoidant coping and lower self-efficacy. They also indicate that high psychological resources do not necessarily buffer the associations linked to disengagement in demanding STEM contexts.

## 1 Introduction

Undergraduate education in science, technology, engineering, and mathematics (STEM) is frequently embedded in high-demand learning environments characterized by intensive workloads, competitive evaluation, and recurring high-stakes assessment. In engineering education, research has described a “stress culture” in which elevated stress is normalized and, in some contexts, implicitly treated as part of academic suc-cess (Jensen and Cross, 2021; Jensen et al., 2023). Extending this work, recent measure-ment studies have operationalized both the stressors students encounter and the extent to which stress is socially normalized within undergraduate engineering contexts (Mirabelli et al., 2025). Qualitative evidence likewise indicates that broader cultural norms in engineering ed-ucation shape how students interpret academic difficulty and their own responses to it, including the meanings they attach to struggle, performance, and belonging (Deters et al., 2024). Research results show that students often face tensions between achievement goals and priorities related to mental health or well-being (Navarro et al., 2025), and meta-analytic evidence indicates that burnout is associated with academic achievement (Madigan and Curran, 2021). Taken together, this literature underscores the need to identify psychological processes that support adaptive adjustment in STEM, particularly processes that may be amenable to educational intervention (Hsu and Goldsmith, 2021).

A particularly consequential psychological resource in academic contexts is self-efficacy. Within social cognitive theory, self-efficacy refers to beliefs about one’s capabilities to organize and execute the actions required to attain desired outcomes (Bandura, 1997). In higher education—and especially in STEM—stronger self-efficacy is consistently associated with greater goal commitment, sustained effort, and persistence in the face of setbacks. Moreover, research in engineering education indicates that expectancy (often operationalized as engineering academic self-efficacy) interacts with task values and perceived costs to predict students’ academic choices, persistence, and performance (Lee et al., 2022).

Converging evidence across STEM learning contexts indicates that self-efficacy is closely tied to continued engagement during challenging problem solving, including self-coaching processes that help students move past metacognitive discomfort (Halmo et al., 2024), and is linked to persistence-related experiences in STEM support contexts (Shortlidge et al., 2024). Importantly, efficacy judgments are influenced not only by past performance but also by how individuals interpret emotional and physiological states during demanding tasks (Bandura, 1997); correspondingly, self-efficacy remains a robust predictor of academic outcomes even when modeled alongside other motivational factors (Von der Mehden et al., 2025).

When students appraise academic demands as stressful, they mobilize coping responses aimed at managing these pressures. According to the transactional model of stress and coping, coping involves the cognitive, emotional, and behavioral efforts individuals use to deal with demands that are perceived as taxing or exceeding their available resources (Lazarus and Folkman, 1984). Among these responses, behavioral disengagement represents an avoidant strategy characterized by withdrawal of effort and the cessation of attempts to address the stressor. Although such strategies may provide short-term emotional relief, meta-analytic evidence indicates that they are generally maladaptive over time in contexts that require sustained engagement and persistence (Suls and Fletcher, 1985).

In STEM learning contexts—where progress typically depends on iterative problem solving, deliberate practice, and feedback—persistent behavioral disengagement may therefore provide immediate relief by lowering task demands while undermining longer-term adjustment by limiting mastery opportunities and weakening engagement. Consistent with this interpretation, behavioral engagement (effort and persistence) shows the strongest association with academic achievement among engagement dimensions in meta-analytic work (Reeve et al., 2025), In STEM instruction, students’ anxiety and perceptions of difficulty can affect performance and persistence (England et al., 2019), while course design and assessment practices can shape students’ stress and anxiety, thereby influencing the contexts in which coping strategies are selected and maintained (Hsu and Goldsmith, 2021).

A central question, then, concerns how behavioral disengagement relates to students’ self-efficacy and through which mechanisms this association may unfold. Emotion-related competencies are likely to be pivotal, given the role of affective cues in efficacy appraisals (Bandura, 1997). Conceptually, emotional competence may *channel* coping patterns into efficacy beliefs: withdrawing effort can reduce opportunities to manage task-related emotions and to receive corrective feedback, thereby diminishing adaptive emotion regulation and, in turn, self-efficacy perceived capability to meet future academic demands.

Contemporary models further emphasize that effective emotion regulation is inherently dynamic: individuals not only choose an initial strategy but also monitor its effectiveness and flexibly maintain or switch strategies as situational demands change (Bonanno and Burton, 2013). Experimental evidence indicates that affective and physiological feedback can guide strategy switching and that such feedback-aligned switching is associated with better adjustment (Birk and Bonanno, 2016). Converging neurocognitive findings show that emotional intensity and initial strategy choice predict switching frequency and that, under high intensity, switching to (or maintaining) distraction is linked to neural indices of greater regulatory success (Dorman Ilan et al., 2019). In demanding STEM contexts, emotional competence may therefore partly reflect the capacity to monitor affective states and flexibly recalibrate regulation in ways that support timely re-engagement with academic challenges.

Resource-based frameworks suggest that the effects of coping on self-efficacy may depend on students’ broader psychological resources. Conservation of Resources theory, stress responses are conceptualized as reactions to threatened or actual resource loss, and resources are understood to cluster into interrelated resource systems that shape individuals’ vulnerability versus resilience (Hobfoll, 1989). From this perspective, hope, resilience, and optimism can be viewed as core, mutually reinforcing resources that support adaptive functioning under pressure (Luthans et al., 2007). In academic contexts, such resources are consistently associated with better adjustment and performance, indicating that students who possess richer internal resource pools may be better equipped to confront stressors (Hazan Liran and Miller, 2019).

Theoretically, hope, resilience, and optimism could buffer the adverse implications of disengagement by sustaining adaptive appraisals and supporting re-engagement; however, it is also plausible that disengagement may be especially consequential among high-resource students if it reflects a sharper self-regulatory breakdown or a greater goal–behavior discrepancy. These competing possibilities motivate explicit tests of conditional (moderated) processes.

Against this background, the present study tests a moderated mediation model (Hayes Model 7) in which behavioral disengagement (X) is associated with self-efficacy (Y) both directly and indirectly through emotional competence (M), while psychological resources—hope, resilience, and optimism (W)—moderate the association between behavioral disengagement and emotional competence (X → M). We hypothesized that behavioral disengagement would be negatively associated with emotional competence and self-efficacy, and emotional competence be positively associated with self-efficacy. We further examined whether the indirect association between behavioral disengagement and self-efficacy via emotional competence varies as a function of psychological resources.

## 2 Materials and methods

### 2.1 Participants

The sample consisted of 162 students, of whom 38.27% were women (*n* = 62), 45.67% were men (*n* = 74), and 16.04% did not report their gender (*n* = 26), aged between 18 and 22 years (*M* = 19.2, SD = 0.75). Regarding the inclusion criteria, participants had among the highest averages on the high school graduation exam (*M* = 9.5), with the maximum grade being 10. The mean age of the women was 19.1 years, and the mean age of the men was 19.2 years.

Participants were eligible if they (a) were undergraduate students enrolled in STEM programs and (b) had high performance on the high school graduation examination, used here as an indicator of prior academic achievement.

### 2.2 Instruments

To characterize coping and personal resources relevant to academic adjustment in STEM, participants completed three self-report instruments: the Psychological Capital Questionnaire (PCQ-12), the Profile of Emotional Competence (PEC), and the Coping Orientation to Problems Experienced (COPE). Together, these measures assess students’ psychological resources in stressful situations and their coping responses.

The PCQ-12, the 12-item short form of the PCQ-24 (Luthans et al., 2007), assesses self-efficacy, optimism, hope, and resilience as indicators of a second-order construct, psychological capital (PsyCap). Items are rated on a 6-point Likert scale (1 = *strongly disagree* to 6 = *strongly agree*) and were adapted to the student context following Hazan Liran and Miller (2019). Higher scores reflect greater PsyCap. In the present sample, internal consistency was good (*α* = 0.851).

The PEC evaluates individual differences in identifying, understanding, expressing, regulating, and using emotions in relation to oneself and others (Brasseur et al., 2013). The 50-item instrument is rated on a 5-point Likert scale and comprises 10 first-order dimensions that load onto two higher-order factors—intrapersonal and interpersonal emotional competence—which together form an overall emotional competence score. The PEC has demonstrated sound psychometric properties across cultural contexts, including Eastern samples (Nozaki et al., 2019). In this study, internal consistency was high (*α* = 0.882).

The COPE Inventory (Carver et al., 1989) assesses 15 coping strategies that can be grouped into four broader categories: problem-focused, emotion-focused, social support–focused, and avoidant coping. Items are rated on a 4-point Likert scale (1 = *I usually do not do this at all* to 4 = *I usually do this to a large extent*). The Romanian validated version was used (Crasovan and Sava, 2013). The present study focused on the behavioral disengagement subscale, conceptualized as an avoidant coping strategy. The overall internal consistency of the COPE in this sample was good (α = 0.841).

### 2.3 Procedure

Participation was voluntary and anonymous. Participants were recruited from first-year STEM cohorts, and eligibility criteria were applied prior to inclusion. Questionnaires were administered in paper-and-pencil format to support standardized administration and quality control during completion.

### 2.4 Analysis

**Figure 1:**
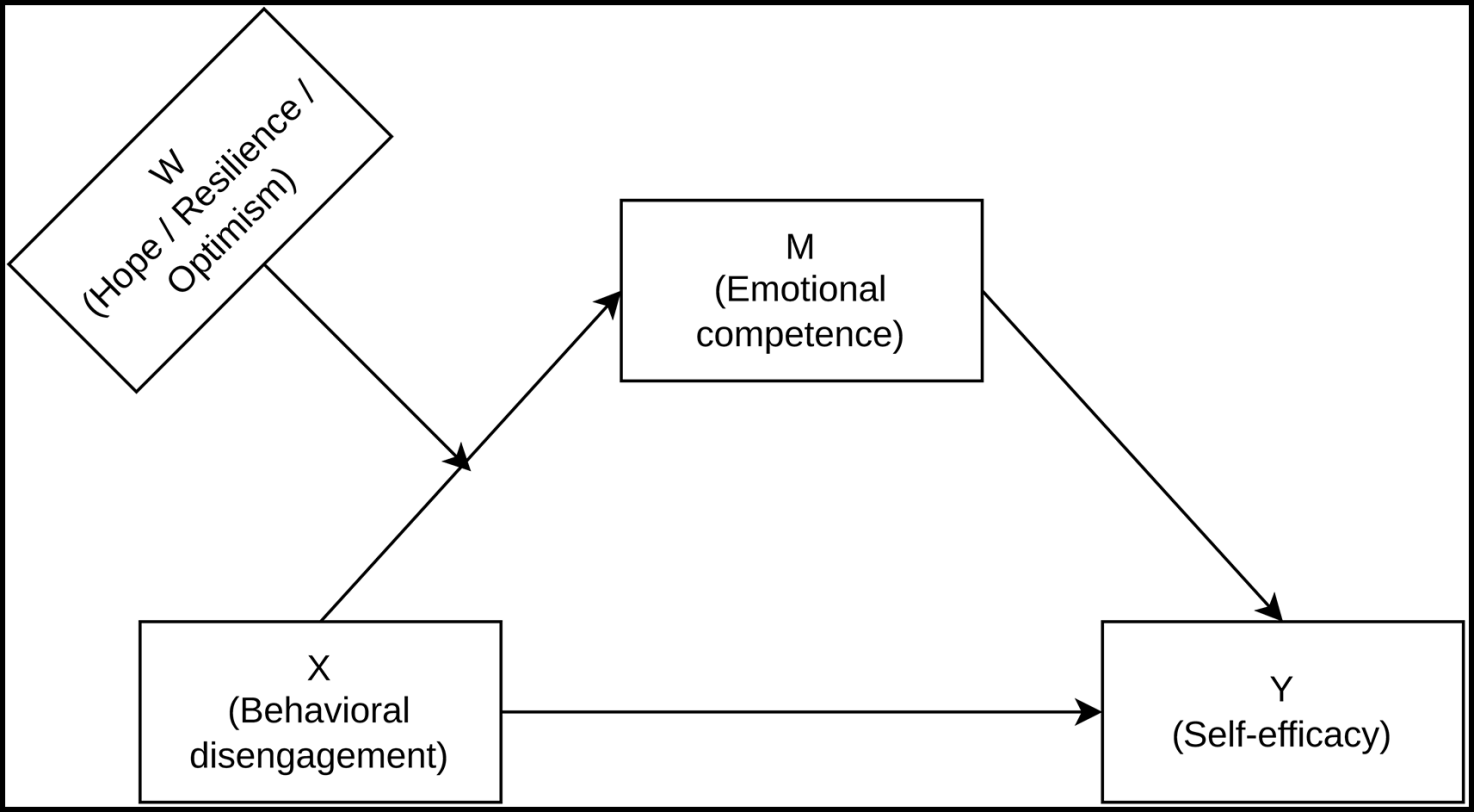
Moderated mediation model (Hayes Model 7). The indirect effect of behavioral disengagement (*X*) on self-efficacy (*Y*) via emotional competence (*M*) is conditional on psychological resources (W: hope/resilience/optimism), which moderate the X → M path.

Analyses were conducted in R (RStudio) using a moderated mediation model (Hayes Model 7), in which the moderator acts on the association between the predictor and the mediator (Hayes, 2017). In line with the study aims, three parallel conditional process models were estimated, each using a different psychological resource (hope, resilience, or optimism) as the moderator (W). Statistical inference was based on *p*-values and 95% confidence intervals interpreted in context rather than on *p*-values alone (Wasserstein and Lazar, 2016). The conceptual model is shown in Figure 1.

Because hope, resilience, and optimism are conceptually related and moderately intercorrelated, an additional robustness analysis was conducted in which all three moderators and their interaction terms with behavioral disengagement were entered simultaneously into the mediator equation, allowing estimation of their unique moderating contributions beyond shared variance.

Missing data were handled using listwise deletion (complete-case analysis; na.omit) on the variables included in each model. From the initial *N* = 162 participants, the analytic sample comprised *N* = 131; 31 cases were excluded due to missing values on covariates (gender: 26 missing; age: 24 missing; 19 missing on both). Missingness was first examined using Little’s MCAR test (Little, 1988) and supplementary logistic missingness analyses. In addition to the complete-case analyses, a sensitivity analysis using multiple imputation by chained equations (20 imputations) was performed using the mice framework (van Buuren and Groothuis-Oudshoorn, 2011). Predictive mean matching was used for age and logistic imputation for gender, and pooled estimates were obtained using Rubin’s rules (Rubin, 1987).

Conditional indirect effects and the index of moderated mediation were estimated via bootstrapping with 5,000 resamples (set.seed(123)), and 95% percentile confidence intervals (CIs) were computed. Bootstrap confidence intervals are recommended for indirect and conditional indirect effects because these quantities often have non-normal sampling distributions (Preacher and Hayes, 2008; Hayes, 2015). Continuous predictors were mean-centered (Xc, Wc), and the interaction term was computed as Xc × Wc. Gender was treated as a factor (female as the reference group); thus, the gender coefficient represents the adjusted mean difference (male − female) in the dependent variable of the respective equation, controlling for the other predictors.

To evaluate whether the final analytic sample was adequate for detecting the moderation component of Hayes’ Model 7, we conducted post-hoc power analyses for the interaction term in the mediator equation (X × W predicting emotional competence). Power was estimated from the incremental variance explained by the interaction term (Δ*R*^2^), expressed as Cohen’s f^2^ (Cohen, 1988), by comparing the full model with a reduced model that omitted the interaction term. Because retrospective power based on observed effects can be misleading, these estimates are reported only as descriptive supplements to the observed effect sizes and confidence intervals (Hoenig and Heisey, 2001).

Collinearity diagnostics indicated low VIF values (approximately 1.00–1.59), which did not suggest substantial collinearity in the estimated models, while recognizing that VIF values are best interpreted contextually rather than through rigid cutoffs (O’Brien, 2007).

## 3 Results

### 3.1 Descriptive statistics and correlations

Skewness and kurtosis values for all continuous variables (including age) were examined descriptively and fell within ranges that did not suggest substantial departures from normality (Kim, 2013). In addition, the use of boostrap confidence intervals reduces reliance on strict normality assumptions for inference regarding indirect and conditional indirect effects. Descriptive statistics for the study variables are summarised in Table 1.

**Table 1:** Descriptive statistics.

Variables	*N*	*M* (SD)	Range (min–max)	Skewness	Kurtosis
B.D.	131	6.65 (2.03)	3.00–15.00	0.87	1.20
E.C.	131	3.51 (0.38)	2.54–4.38	−0.12	−0.51
Self-efficacy	131	13.47 (2.76)	4.00–18.00	−0.69	0.42
Hope	131	18.17 (2.58)	11.00–24.00	−0.21	0.23
Resilience	131	13.27 (2.80)	6.00–18.00	−0.24	−0.42
Optimism	131	9.49 (1.99)	2.00–12.00	−0.68	0.18
Age	131	19.18 (0.75)	18.00–22.00	1.41	2.84
Gender	131	0.52 (0.50)	0.00–1.00	–	–

B.D., Behavioral Disengagement, E.C., Emotional Competence. Gender was coded 0 = female, 1 = male.

As shown in Table 2, the moderators (Hope / Resilience / Optimism) were positively correlated with emotional competence and self-efficacy (e.g., Hope: *r* = 0.57, *p* < 0.001; Resilience: *r* = 0.61, *p* < 0.001) and showed moderate intercorrelations. Behavioral disengagement was negatively correlated with emotional competence (*r* = −0.29, *p* < 0.001) and with self-efficacy (*r* = −0.27, *p* < 0.01), whereas emotional competence was positively associated with self-efficacy (*r* = 0.53, *p* < 0.001).

**Table 2:** Pearson correlations.

Variables	B.D.	E.C.	Self-efficacy	Hope	Resilience	Optimism	Age	Gender
B.D.	–							
E.C.	−0.29***	–						
Self-efficacy	−0.27**	0.53***	–					
Hope	−0.17	0.49***	0.57***	–				
Resilience	−0.18*	0.51***	0.61***	0.46***	–			
Optimism	−0.22*	0.41***	0.40***	0.31***	0.41***	–		
Age	0.05	0.21*	0.13	0.08	0.00	−0.05	–	
Gender	0.03	0.04	0.35***	0.13	0.34***	0.09	0.05	–

B.D., Behavioral Disengagement, E.C., Emotional Competence; *p < 0.05, **p < 0.01, ***p < 0.001. Gender was coded 0 = female, 1 = male.

### 3.2 Moderated mediation analyses

A moderated mediation model was tested in which behavioral disengagement predicted self-efficacy through emotional competence, with the moderator influencing the X → M path. Three separate models were run, using Hope, Resilience, and Optimism, respectively, as the moderator.

Post-hoc power analyses indicated that the interaction effects in the mediator equation were small (Hope: Δ*R*^2^ = 0.022, *f*^2^ = 0.033, power = 0.530; Resilience: Δ*R*^2^ = 0.026, *f*^2^ = 0.042, power = 0.631; Optimism: Δ*R*^2^ = 0.025, *f*^2^ = 0.035, power = 0.549). Following recommendations that retrospective power should be interpreted cautiously, these estimates are reported only as descriptive indicators of the sample’s limited sensitivity to small interaction effects, alongside the observed effect sizes and confidence intervals (Cohen, 1988; Hoenig and Heisey, 2001).

Missing data were confined to the covariates age (24 missing values; 14.81%) and gender (26 missing values; 16.05%), with 19 cases missing on both variables, resulting in 31 incomplete cases and 131 complete cases. Little’s MCAR test was significant, *χ*^2^(20) = 39.82, *p* = 0.005, indicating that the data were not consistent with a Missing Completely at Random mechanism. Supplementary logistic missingness analyses further suggested that missingness in gender was associated with hope (*B* = −0.301, *p* = 0.003), whereas missingness in age showed marginal associations with hope (B = −0.179, *p* = 0.065) and resilience (*B* = −0.190, *p* = 0.075). These findings suggest that complete-case analysis may have reduced power and may have introduced bias.

To assess robustness, the models were re-estimated using multiple imputation (*m* = 20), preserving the full sample of *N* = 162. The substantive pattern of results was broadly similar to the complete-case analyses. In the mediator equation, the interaction term remained significant for Hope (*B* = −0.007, *p* = 0.007) and Resilience (*B* = −0.009, *p* = 0.003), whereas the interaction for Optimism was attenuated and no longer reached the conventional significance threshold (*B* = −0.010, *p* = 0.073). In the outcome equation, emotional competence remained a significant predictor of self-efficacy across all three models. Overall, these results suggest that the main pattern of moderated mediation was reasonably robust, although the optimism model appeared more sensitive to the handling of missing data.

#### 3.2.1 Hope as a moderator

**Figure 2:**
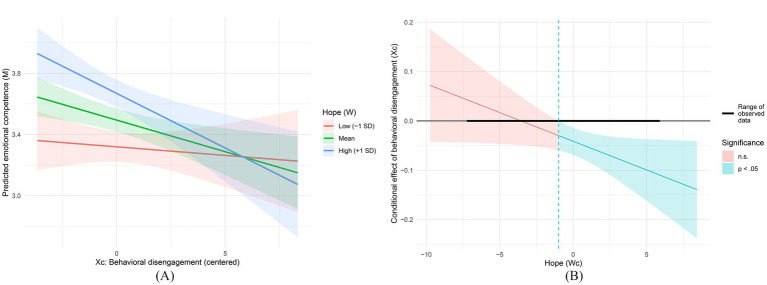
**(A)** Johnson–Neyman plot probing the Xc × Wc interaction: conditional effect (slope) of behavioral disengagement on emotional competence across values of hope; shaded regions denote statistically non-significant versus significant (*p* < 0.05) slopes, and the horizontal bar indicates the observed range of the moderator in the sample. **(B)** Interaction plot for the mediator equation: predicted emotional competence (M) as a function of behavioral disengagement (Xc) at low (−1 SD), mean, and high (+1 SD) levels of hope; shaded bands indicate 95% confidence intervals.

In the mediator equation, behavioral disengagement negatively predicted emotional competence (*B* = −0.041, *p* = 0.003, 95% CI [−0.069, −0.014]), whereas hope positively predicted emotional competence (*B* = 0.068, *p* < 0.001, 95% CI [0.046, 0.089]). The behavioral disengagement × hope interaction was significant (*B* = −0.012, *p* = 0.044, 95% CI [−0.023, 0.000]), indicating that the negative association between behavioral disengagement and emotional competence became stronger at higher levels of hope; however, because the confidence interval approached zero at its upper bound, this interaction should be interpreted cautiously. The mediator model explained 34.1% of the variance in emotional competence (*R*^2^ = 0.341). The results are reported in Table 3 and illustrated in Figure 2.

**Table 3:** Mediator equation with hope as a moderator.

Predictor	*B*	*SE*	*t*	*p*	95% CI [LL, UL]
Intercept	1.695	0.703	2.41	0.017	[0.303, 3.088]
B.D. (Xc)	−0.041	0.014	−2.99	0.003	[−0.069, −0.014]
Hope (Wc)	0.068	0.011	6.14	<0.001	[0.046, 0.089]
Xc × Wc	−0.012	0.006	−2.03	0.044	[− 0.023, 0.000]
Age	0.094	0.037	2.56	0.012	[0.021, 0.167]
Gender	−0.005	0.055	−0.09	0.929	[−0.115, 0.105]
*R* ^2^	0.341				

B.D., Behavioral disengagement. B, unstandardized regression coefficient; SE, standard error; CI, confidence interval; LL, lower limit; UL, upper limit. Predictors were mean centered (Xc, Wc), and Xc × Wc represents the interaction. Gender was entered as a factor (reference = female).

In the outcome equation, emotional competence positively predicted self-efficacy (*B* = 2.151, *p* < 0.001, 95% CI [1.055, 3.246]), and the direct effect of behavioral disengagement on self-efficacy, controlling for the mediator, the moderator, and the covariates, remained negative and met the conventional 0.05 criterion (*B* = −0.184, *p* = 0.043, 95% CI [−0.361, −0.006]). Hope positively predicted self-efficacy (*B* = 0.393, *p* < 0.001, 95% CI [0.240, 0.547]). Gender (men relative to women) was associated with higher self-efficacy (*B* = 1.613, *p* < 0.001, 95% CI [0.925, 2.302]). The outcome model explained 51.1% of the variance in self-efficacy (*R*^2^ = 0.511). The results are reported in Table 4.

**Table 4:** Outcome equation predicting self-efficacy: hope as a moderator.

Predictor	*B*	*SE*	*t*	*p*	95% CI [LL, UL]
Intercept	2.877	4.523	0.64	0.526	[−6.075, 11.829]
B.D. (Xc)	−0.184	0.090	−2.05	0.043	[−0.361, −0.006]
E.C. (M)	2.151	0.554	3.88	<0.001	[1.055, 3.246]
Hope (Wc)	0.393	0.077	5.08	<0.001	[0.240, 0.547]
Age	0.115	0.237	0.48	0.629	[−0.354, 0.583]
Gender	1.613	0.348	4.64	<0.001	[0.925, 2.302]
*R* ^2^	0.511				

B.D., Behavioral Disengagement, E.C., Emotional Competence. B, unstandardized regression coefficient; SE, standard error; CI, confidence interval; LL, lower limit; UL, upper limit. Xc and Wc are mean-centered. Gender was entered as a factor (reference = female); the coefficient represents the adjusted mean difference (male–female).

**Figure 3:**
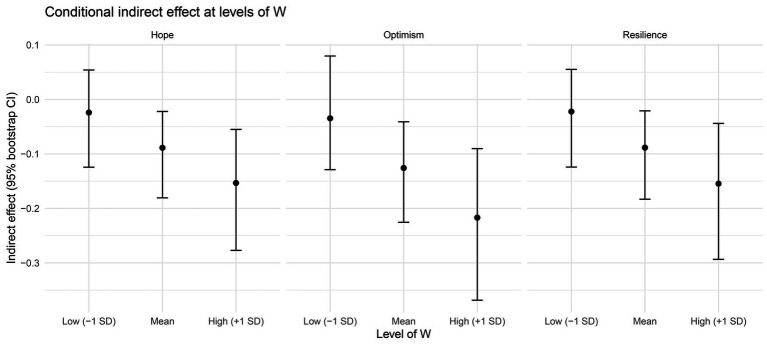
Conditional indirect effects of behavioral disengagement on self-efficacy through emotional competence at low (−1 SD), mean, and high (+1 SD) levels of hope, optimism, and resilience. Indirect effects were estimated using bootstrap resampling (5,000 samples); error bars represent 95% bootstrap confidence intervals.

The conditional indirect effect of behavioral disengagement on self-efficacy through emotional competence was non-significant at a low level of hope (−1 SD, ab = −0.024, 95% CI [−0.124, 0.054]), but significant at the mean level (ab = −0.089, 95% CI [−0.181, −0.022]) and at the high level (+1 SD, ab = −0.153, 95% CI [−0.277, −0.055]). The results are reported in Table 5 and illustrated in Figure 3.

**Table 5:** Bootstrapped conditional indirect effects of behavioral disengagement on self-efficacy via emotional competence across levels of hope.

Moderator (W)	Level of W	Indirect effect (ab)	95% bootstrap CI [LL, UL]
Hope	Low (−1 SD)	−0.024	[−0.124, 0.054]
Hope	Mean	−0.089	[−0.181, −0.022]
Hope	High (+1 SD)	−0.153	[−0.277, −0.055]

SD, standard deviation; LL, lower limit; UL, upper limit. Indirect effect (ab), conditional indirect effect of X on Y through M. Bootstrap confidence intervals are based on 5,000 resamples (percentile 95% CI).

The index of moderated mediation (IMM) was significant (IMM = −0.025, 95% CI [−0.051, −0.004]). The results are shown in Table 6.

**Table 6:** Index of moderated mediation for the conditional indirect effect.

Moderator (W)	IMM (a₃b)	95% bootstrap CI [LL, UL]
Hope	−0.025	[−0.051, −0.004]

LL, lower limit; UL, upper limit; IMM, index of moderated mediation (a₃b). Percentile bootstrap 95% confidence intervals are based on 5,000 resamples.

#### 3.2.2 Resilience as a moderator

**Figure 4:**
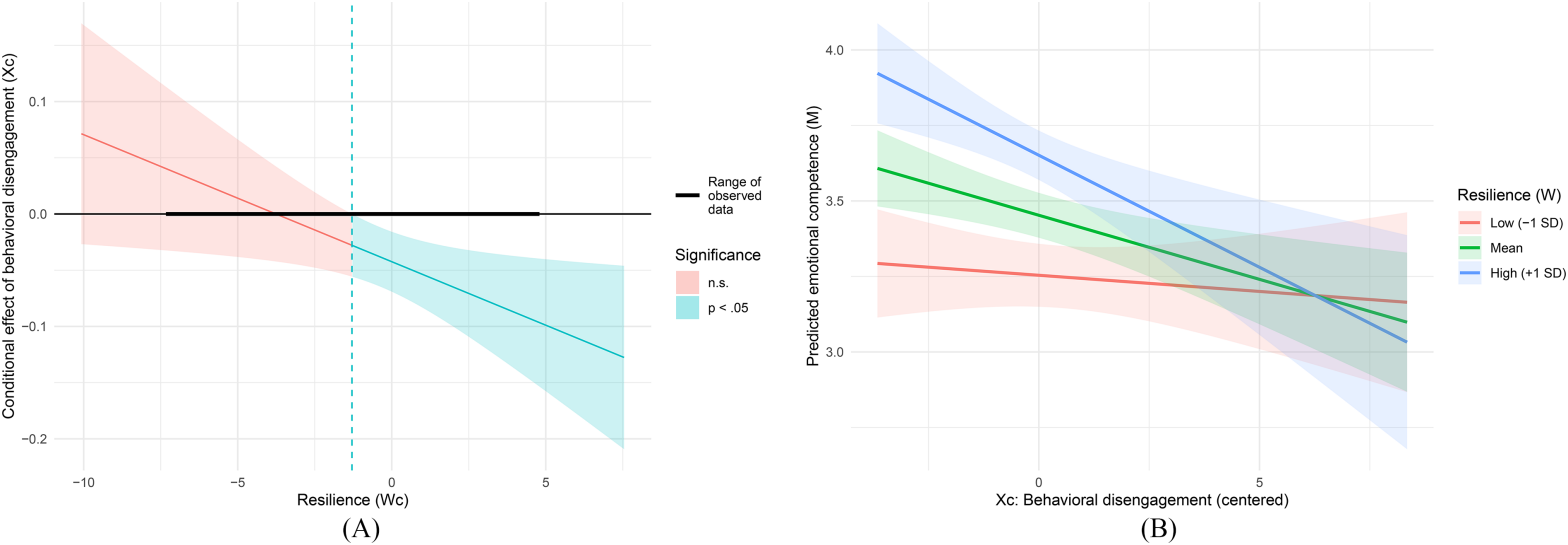
**(A)** Johnson–Neyman plot probing the Xc × Wc interaction: conditional effect (slope) of behavioral disengagement (Xc) on emotional competence across values of resilience; shaded regions denote statistically non-significant versus significant (*p* < 0.05) slopes, and the horizontal bar indicates the observed range of the moderator in the sample. **(B)** Interaction plot for the mediator equation: predicted emotional competence (M) as a function of behavioral disengagement (Xc) at low (−1 SD), mean, and high (+1 SD) levels of resilience (Wc); shaded bands indicate 95% confidence intervals.

In the mediator equation, behavioral disengagement negatively predicted emotional competence (*B* = −0.042, *p* = 0.002, 95% CI [−0.069, −0.016]), whereas resilience positively predicted emotional competence (*B* = 0.071, *p* < 0.001, 95% CI [0.051, 0.091]). The interaction between behavioral disengagement and resilience was significant (*B* = −0.011, *p* = 0.023, 95% CI [−0.021, −0.002]), indicating that the negative X → M association becomes stronger at higher levels of resilience. The mediator model explained 38.8% of the variance in emotional competence (*R*^2^ = 0.388). The results are reported in Table 7 and illustrated in Figure 4.

**Table 7:** Mediator equation with resilience as a moderator.

Predictor	*B*	*SE*	*t*	*p*	95% CI [LL, UL]
Intercept	1.352	0.676	2.00	0.048	[0.015, 2.689]
B.D (Xc)	−0.042	0.013	−3.15	0.002	[−0.069, −0.016]
Resilience (Wc)	0.071	0.010	6.93	<0.001	[0.051, 0.091]
Xc × Wc	−0.011	0.005	−2.29	0.023	[−0.021, −0.002]
Age	0.114	0.035	3.24	0.002	[0.044, 0.184]
Gender	−0.090	0.057	−1.60	0.113	[−0.203, 0.022]
*R* ^2^	0.388				

B.D., Behavioral disengagement. B, unstandardized regression coefficient; SE, standard error; CI, confidence interval; LL, lower limit; UL, upper limit. Predictors were mean centered (Xc, Wc), and Xc × Wc represents the interaction. Gender was entered as a factor (reference = female).

In the outcome equation, emotional competence positively predicted self-efficacy (*B* = 2.084, *p* < 0.001, 95% CI [0.933, 3.234]), and the direct effect of behavioral disengagement on self-efficacy remained negative and narrowly met the conventional 0.05 criterion (*B* = −0.181, *p* = 0.049, 95% CI [−0.362, −0.001]). Resilience positively predicted self-efficacy (*B* = 0.361, *p* < 0.001, 95% CI [0.205, 0.517]). Gender (men relative to women) was associated with higher self-efficacy (*B* = 1.184, *p* = 0.002, 95% CI [0.434, 1.933]). The outcome model explained 49.5% of the variance in self-efficacy (*R*^2^ = 0.495). The results are shown in Table 8.

**Table 8:** Outcome equation predicting self-efficacy: resilience as a moderator.

Predictor	*B*	*SE*	*t*	*p*	95% CI [LL, UL]
Intercept	1.054	4.554	0.23	0.817	[−7.959, 10.067]
B.D (Xc)	−0.181	0.091	−1.99	0.049	[−0.362, −0.001]
E.C. (M)	2.084	0.581	3.58	<0.001	[0.933, 3.234]
Resilience (Wc)	0.361	0.079	4.58	<0.001	[0.205, 0.517]
Age	0.234	0.243	0.98	0.338	[−0.247, 0.714]
Gender	1.184	0.379	3.12	0.002	[0.434, 1.933]
*R* ^2^	0.495				

B.D., Behavioral Disengagement, E.C., Emotional Competence. B, unstandardized regression coefficient; SE, standard error; CI, confidence interval; LL, lower limit; UL, upper limit. Xc and Wc are mean-centered. Gender was entered as a factor (reference = female); the coefficient represents the adjusted mean difference (male–female).

The conditional indirect effect of behavioral disengagement on self-efficacy through emotional competence was non-significant at low resilience (−1 SD, ab = −0.022, 95% CI [−0.124, 0.055]), but significant at the mean level (ab = −0.088, 95% CI [−0.183, −0.021]) and at high resilience (+1 SD, ab = −0.155, 95% CI [−0.294, −0.044]). The results are reported in Table 9 and illustrated in Figure 3.

**Table 9:** Bootstrapped conditional indirect effects of behavioral disengagement on self-efficacy via emotional competence across levels of resilience.

Moderator (W)	Level of W	Indirect effect (ab)	95% bootstrap CI [LL, UL]
Resilience	Low (−1 SD)	−0.022	[−0.124, 0.055]
Resilience	Mean	−0.088	[−0.183, −0.021]
Resilience	High (+1 SD)	−0.155	[−0.294, −0.044]

SD, standard deviation; LL, lower limit; UL, upper limit. Indirect effect (ab), conditional indirect effect of X on Y through M. Bootstrap confidence intervals are based on 5,000 resamples (percentile 95% CI).

As shown in Table 10, the index of moderated mediation was significant (IMM = *−*0.024, 95% CI [−0.051, −0.002]).

**Table 10:** Index of moderated mediation for the conditional indirect effect.

Moderator (W)	IMM (a₃b)	95% bootstrap CI [LL, UL]
Resilience	−0.024	[−0.051, −0.002]

LL, lower limit; UL, upper limit; IMM, index of moderated mediation (a₃b). Percentile bootstrap 95% confidence intervals are based on 5,000 resamples.

#### 3.2.3 Optimism as a moderator

**Figure 5:**
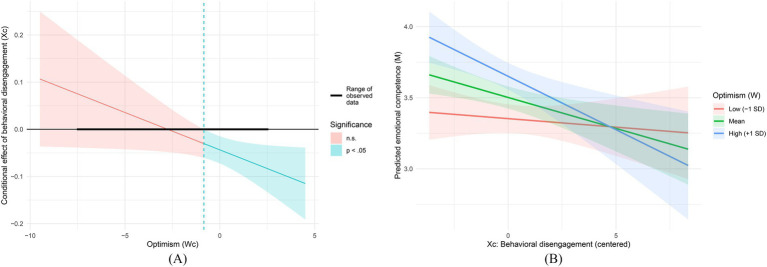
**(A)** Johnson-Neyman plot probing the Xc × Wc interaction: conditional effect (slope) of behavioral disengagement on emotional competence across values of optimism; shaded regions denote statistically non-significant versus significant (*p* < 0.05) slopes, and the horizontal bar indicates the observed range of the moderator in the sample. **(B)** Interaction plot for the mediator equation: predicted emotional competence (M) as a function of behavioral disengagement (Xc) at low (−1 SD), mean, and high (+1 SD) levels of optimism (Wc); shaded bands indicate 95% confidence intervals.

In the mediator equation, behavioral disengagement negatively predicted emotional competence (*B* = −0.044, *p* = 0.003, 95% CI [−0.072, −0.015]), whereas optimism positively predicted emotional competence (*B* = 0.075, *p* < 0.001, 95% CI [0.045, 0.104]). The interaction between behavioral disengagement and optimism was significant (*B* = −0.016, *p* = 0.039, 95% CI [−0.031, −0.001]), indicating the same pattern: the negative X → M association is stronger at higher levels of optimism. The mediator model explained 29.0% of the variance in emotional competence (*R*^2^ = 0.290). The results are reported in Table 11 and illustrated in Figure 5.

**Table 11:** Mediator equation with optimism as a moderator.

Predictor	*B*	*SE*	*t*	*p*	95% CI [LL, UL]
Intercept	1.306	0.734	1.85	0.066	[−0.093, 2.813]
B.D (Xc)	−0.044	0.015	−3.00	0.003	[−0.072, −0.015]
Optimism (Wc)	0.075	0.015	5.02	<0.001	[0.045, 0.104]
Xc × Wc	−0.016	0.008	−2.08	0.039	[−0.031, −0.001]
Age	0.111	0.038	2.88	0.005	[0.035, 0.187]
Gender	0.020	0.058	0.34	0.733	[−0.095, 0.134]
*R* ^2^	0.290				

B.D., Behavioral disengagement. B, unstandardized regression coefficient; SE, standard error; CI, confidence interval; LL, lower limit; UL, upper limit. Predictors were mean centered (Xc, Wc), and Xc × Wc represents the interaction. Gender was entered as a factor (reference = female).

In the outcome equation, emotional competence positively predicted self-efficacy (*B* = 2.888, *p* < 0.001, 95% CI [1.758, 4.017]). The direct effect of behavioral disengagement on self-efficacy was negative but did not reach the conventional 0.05 significance threshold (B = −0.171, *p* = 0.078, 95% CI [−0.363, 0.020]). Optimism positively predicted self-efficacy (*B* = 0.264, *p* = 0.012, 95% CI [0.058, 0.469]). Gender (men relative to women) was associated with higher self-efficacy (*B* = 1.752, *p* < 0.001, 95% CI [1.017, 2.487]). The outcome model explained 43.9% of the variance in self-efficacy (*R*^2^ = 0.439). The results are shown in Table 12.

**Table 12:** Outcome equation predicting self-efficacy: optimism as a moderator.

Predictor	*B*	*SE*	*t*	*p*	95% CI [LL, UL]
Intercept	−0.846	4.777	−0.18	0.860	[−10.299, 8.608]
B.D (Xc)	−0.171	0.097	−1.78	0.078	[−0.363, 0.020]
E.C. (M)	2.888	0.571	5.06	<0.001	[1.758, 4.017]
Optimism (Wc)	0.264	0.104	2.54	0.012	[0.058, 0.469]
Age	0.170	0.256	0.66	0.508	[−0.337, 0.677]
Gender	1.752	0.371	4.72	<0.001	[1.017, 2.487]
*R* ^2^	0.439				

B.D., Behavioral Disengagement; E.C., Emotional Competence. B, unstandardized regression coefficient; SE, standard error; CI, confidence interval; LL, lower limit; UL, upper limit. Xc and Wc are mean-centered. Gender was entered as a factor (reference = female); the coefficient represents the adjusted mean difference (male–female).

The conditional indirect effect of behavioral disengagement on self-efficacy through emotional competence was non-significant at low optimism (−1 SD, ab = −0.035, 95% CI [−0.129, 0.080]), but significant at the mean level (ab = −0.126, 95% CI [−0.226, −0.041]) and at high optimism (+1 SD, ab = −0.217, 95% CI [−0.368, −0.090]). The results are reported in Table 13 and illustrated in Figure 3.

**Table 13:** Bootstrapped conditional indirect effects of behavioral disengagement on self-efficacy via emo-tional competence across levels of optimism.

Moderator (W)	Level of W	Indirect effect (ab)	95% bootstrap CI [LL, UL]
Optimism	Low (−1 SD)	−0.035	[−0.129, 0.079]
Optimism	Mean	−0.126	[−0.226, −0.041]
Optimism	High (+1 SD)	−0.217	[−0.368, −0.090]

SD, standard deviation; LL, lower limit; UL, upper limit. Indirect effect (ab), conditional indirect effect of X on Y through M. Bootstrap confidence intervals are based on 5,000 resamples (percentile 95% CI).

The index of moderated mediation was significant (IMM = −0.046, 95% CI [−0.090, −0.011]). The results are shown in Table 14.

**Table 14:** Index of moderated mediation for the conditional indirect effect.

Moderator (W)	IMM (a₃b)	95% bootstrap CI [LL, UL]
Optimism	−0.046	[−0.090, −0.011]

LL, lower limit; UL, upper limit; IMM, index of moderated mediation (a₃b). Percentile bootstrap 95% confidence intervals are based on 5,000 resamples.

To examine whether the moderation effects reflected unique contributions of hope, resilience, and optimism or variance shared among these related psychological capital components, an additional combined model was estimated including all three moderators and their interaction terms with behavioral disengagement simultaneously. In this model, none of the interaction terms remained statistically significant (Hope: *B* = −0.004, *p* = 0.513; Resilience: *B* = −0.007, *p* = 0.219; Optimism: *B* = −0.003, *p* = 0.674). At the same time, the main effects of hope, resilience, and optimism on emotional competence remained positive and significant. These findings suggest that the moderation effects observed in the separate models may partly reflect shared variance among the psychological capital components rather than fully distinct moderating effects.

Although this pattern is consistent with the interpretation that the separate moderation effects may partly reflect shared variance among the psychological capital components, it should not be interpreted exclusively in that way. Given the modest sample size and the inclusion of multiple interaction terms in the same model, the combined analysis may also have had limited power to detect small unique moderation effects. In addition, conceptual overlap among the moderators may have reduced the precision of the interaction estimates, even though conventional collinearity diagnostics did not indicate severe multicollinearity.

As an exploratory follow-up, we also tested a composite psychological capital score derived from hope, resilience, and optimism. In this model, the interaction between behavioral disengagement and the PsyCap composite significantly predicted emotional competence (*B* = −0.038, *p* = 0.031), while emotional competence remained a significant predictor of academic self-efficacy (*B* = 1.240, *p* = 0.036), and the PsyCap composite showed a strong positive association with self-efficacy (*B* = 1.795, *p* < 0.001). The internal consistency of the exploratory composite score was modest (Cronbach’s *α* = 0.66), suggesting that the index may capture a shared positive-resource dimension, although the three components should not be treated as fully interchangeable.

## 4 Discussion

The present study tested a moderated mediation model in which behavioral disengagement was associated with self-efficacy both directly and indirectly via emotional competence, while psychological resources (hope, resilience, optimism) conditioned the association between behavioral disengagement and emotional competence. At the bivariate level, correlations were consistent with the hypothesized pattern: behavioral disengagement was negatively correlated with emotional competence and self-efficacy, whereas emotional competence was positively associated with self-efficacy.

Multivariate analyses revealed a robust pattern across all three models. First, behavioral disengagement negatively predicted emotional competence. Second, each psychological resource (hope, resilience, and optimism) positively predicted emotional competence. Third, the interaction terms (behavioral disengagement × psychological resource) were significant in all three model variants, indicating that the association between behavioral disengagement and emotional competence depends on the level of the psychological resource. In the outcome equation, emotional competence consistently and positively predicted self-efficacy, and each moderator also had a positive main effect on self-efficacy.

Although the present model was theoretically grounded in the literature on coping, emotional regulation, and academic self-efficacy, alternative model configurations are also plausible, particularly given the cross-sectional design and the conceptual relatedness of the constructs examined. For instance, psychological resources such as hope, resilience, and optimism could also be conceptualized as antecedents of self-efficacy operating through emotional competence, or emotional competence itself could be examined as a moderator rather than a mediator. Because the data were cross-sectional, the present study cannot adjudicate between these alternative structural possibilities. Accordingly, the proposed model should be interpreted as one theoretically informed representation of the associations among these variables rather than the only plausible explanatory framework. Future research would benefit from longitudinal designs and comparative model-testing approaches to evaluate alternative pathways more rigorously.

### 4.1 Emotional competence in the association between coping and self-efficacy

The stable, positive association between emotional competence and self-efficacy supports the view that socio-cognitive resources (identifying, understanding, and regulating emotions in relation to self and others) are linked to stronger perceived capability to cope with academic demands. Functionally, emotional competence may facilitate more adaptive appraisal of academic difficulties, sustained effort, and flexible strategy adjustment under stress, thereby translating into higher self-efficacy even after controlling for behavioral disengagement, the psychological resource, and covariates. This finding underscores the role of socio-cognitive competencies—identifying, understanding, and regulating emotions—in shaping students’ perceived capacity to cope with academic demands.

Consistent with this pattern, conditional indirect effects were significant at medium and high levels of each moderator, suggesting that the association between behavioral disengagement and self-efficacy is partly explained by emotional competence, but not uniformly across participants, rather, it varies as a function of psychological resource levels.

Functionally, higher emotional competence may allow students to adaptively appraise challenges, maintain effort under stress, and flexibly adjust strategies, thereby supporting self-efficacy. Importantly, conditional indirect effects revealed that this indirect association was evident selectively, becoming significant at medium and high levels of psychological resources, highlighting that the association involving behavioral disengagement depends on the broader resource context.

### 4.2 Moderation patterns: amplification rather than buffering

A notable finding concerns the direction of moderation: the consistently negative X x W coefficients indicate that the negative association between behavioral disengagement and emotional competence becomes stronger at higher levels of hope, resilience, and optimism. In other words, the psychological resources examined did not operate as “buffers” of the X → M association in these data; instead, they amplified the negative slope. This pattern is further supported by the conditional indirect effects, which were non-significant at low levels of the moderators but became significant and more negative at medium and high levels, alongside significant indices of moderated mediation in all three models.

In other words, that psychological resources did not buffer the negative effect of disengagement on emotional competence; rather, higher levels of hope, resilience, and optimism amplified the negative association. Specifically, the X × W interactions were consistently negative, and conditional indirect effects became more negative at higher moderator levels.

One plausible interpretation worth discussing in relation to prior evidence is that, in demanding academic contexts, high psychological resources may co-occur with higher personal standards, stronger goal orientation, and greater performance-related investment. Under such conditions, the emergence of behavioural disengagement may be particularly “costly” for socio-emotional functioning (e.g., withdrawal from effort may be accompanied by cognitive dissonance, rumination, or reduced emotion-regulation capacity), resulting in a sharper decline in self-reported emotional competence. Alternatively, at high levels of psychological resources, behavioural disengagement may reflect more abrupt, “all-or-nothing” episodes of disengagement, with disproportionately negative consequences for emotional processing. An alternative explanation is that students with higher psychological resources may also be more self-aware and more candid in reporting reduced emotional competence when they disengage. Thus, the stronger negative association observed at higher resource levels may partly reflect response tendencies linked to greater self-awareness and more accurate self-appraisal in self-report data, rather than only a genuine psychological decrement. These accounts would benefit from direct comparison with prior findings and, ideally, from longitudinal designs capable of distinguishing transient vulnerability from stable individual differences.

So, potential explanations include:

1. Students with high psychological resources may hold higher personal standards and stronger goal orientation, making disengagement particularly discordant and stressful.
2. Behavioral disengagement among high-resource students may reflect abrupt or extreme withdrawal episodes, with disproportionate consequences for emotional processing and self-regulation.

This pattern suggests that high-resource students are not immune to the costs of avoidant coping, which has important implications for both research and educational practice.

This pattern may indicate that the buffering role identified in the separate models is better understood at the level of shared positive psychological resources than as a uniquely specific function of hope, resilience, or optimism taken in isolation.

At the same time, the absence of significant unique interaction terms in the combined model should be interpreted cautiously. Although this pattern is consistent with shared variance among hope, resilience, and optimism, it may also reflect the more demanding nature of a model that simultaneously includes several related moderators and interaction terms. In the present study, conventional collinearity diagnostics did not indicate severe multicollinearity, but limited statistical power and reduced precision for small unique interaction effects remain plausible alternative explanations.

The exploratory composite-model findings further support the possibility that hope, resilience, and optimism may function less as fully independent moderators and more as interrelated components of a broader positive psychological resource, consistent with the notion of psychological capital. Notably, whereas the unique interaction terms were no longer significant when all three moderators were entered simultaneously, the interaction involving their composite score remained significant. This pattern suggests that the moderation process may be located more strongly at the level of shared psychological capital than at the level of sharply differentiated resource-specific effects. However, this exploratory composite should be interpreted cautiously. Its internal consistency was modest (*α* = 0.66), which suggests some shared variance among the three resources, but also indicates that they are not fully redundant. Accordingly, the present findings are more consistent with the idea of partially integrated positive psychological resources than with the assumption that the three components represent a single homogeneous construct. Future research should test higher-order and latent-variable models of psychological capital more directly.

### 4.3 Differences across models and implications for the nature of mediation

In the hope and resilience models, the direct effect of behavioral disengagement on self-efficacy remained significant, supporting partial mediation: emotional competence accounts for part of the X-Y relationship, while a direct component remains, pointing to additional mechanisms (e.g., motivational processes, self-regulation, fatigue/sustained effort, learning strategies). In the optimism model, the direct effect was negative but did not reach the conventional 0.05 threshold, while conditional indirect effects and the index remained significant at medium and high optimism. So, in the optimism model, the direct effect was marginally non-significant, suggesting that emotional competence may transmit a larger portion of the disengagement effect under high optimism.

### 4.4 Covariates and relevance for the studied population

The positive association of gender (men relative to women) with self-efficacy was consistent across all models, indicating systematic differences that warrant discussion in relation to research on academic socialization and self-evaluations of competence. So, gender consistently predicted self-efficacy, with men reporting higher levels than women, aligning with prior research on academic socialization and self-evaluations in STEM. Age predicted emotional competence but not self-efficacy, suggesting developmental gains in emotional processing within this relatively narrow age range. These findings reinforce the need to consider demographic and developmental factors in coping and efficacy research.

### 4.5 Practical implications

From an educational standpoint, the findings point to two complementary directions: (1) reducing behavioural disengagement (through engagement-oriented interventions, study/learning strategies, and self-regulation training) and (2) strengthening emotional competence as a resource robustly linked to self-efficacy. Moreover, the moderation pattern suggests that students with high psychological resources are not necessarily “immune” to the costs of disengagement; on the contrary, when disengagement occurs, its socio-emotional consequences may be more pronounced. This highlights the value of early screening and prevention of disengagement even among apparently “resilient” groups.

### 4.6 Limitation and future research

First, limitation concerns missing data. Approximately 19% of the initial sample was lost under complete-case analysis due to missing values on age and gender, which reduced the effective sample size from 162 to 131. The missing data analyses indicated that the missingness pattern was not fully consistent with MCAR, suggesting that listwise deletion may have reduced statistical power and may also have introduced bias. Although multiple imputation analyses yielded a broadly similar overall pattern of findings, some effects—particularly those involving optimism—were attenuated, indicating that parts of the model may be sensitive to the method used to handle missing data.

Another concern, the final sample size (*N* = 131) was relatively small for detecting interaction effects in a moderated mediation framework. Post-hoc power analyses showed that achieved power for the observed interaction terms was below the conventional 0.80 threshold, indicating that the study may have been underpowered for weaker moderation effects. This should be considered when interpreting the findings and their generalizability. In addition, the modest final sample size may have affected the stability and replicability of the moderation estimates, particularly for weaker interaction effects.

The exclusive use of self-report measures raises the possibility of common method variance and shared-method inflation of associations among the variables. This should be regarded as a core limitation of the study. Future research would benefit from incorporating multi-method assessments, such as performance-based indicators, peer or instructor reports, and behavioral indicators of academic disengagement, in order to strengthen convergent validity and reduce method-related bias.

A final limitation concerns the fact that hope, resilience, and optimism were initially tested in separate moderated mediation models. Although this strategy was useful for examining each resource individually, an additional combined model showed that the interaction terms were no longer significant when the three moderators were entered simultaneously. This suggests that the observed moderation effects may reflect shared variance among related psychological capital components, limiting conclusions about the unique moderating role of each specific resource. In addition, since the combined model included several related moderators and interaction terms, the absence of significant unique interaction effects may also partly reflect limited power and reduced precision rather than only substantive overlap among the constructs. The exploratory PsyCap composite findings point to the possibility that these resources operate in a partially integrated manner, but this interpretation remains provisional and should be tested more rigorously in larger samples using latent-variable approaches.

## 5 Conclusion

The present study advances the understanding of self-efficacy as a socio-cognitive outcome associated with the dynamic interaction between maladaptive coping, emotional competence, and positive psychological resources. By testing a moderated mediation framework, findings suggest that behavioral disengagement is not merely directly associated with self-efficacy but is also indirectly associated with it through emotional competence under specific conditions. This indirect pathway was conditional on levels of hope, resilience, and optimism, which moderate the association between disengagement and emotional competence.

Across all three models examined in this study, the indirect effect of behavioural disengagement on self-efficacy through emotional competence was non-significant at low levels of psychological resources but became increasingly negative at moderate and high levels. This pattern suggests that emotional competence is linked to the association between disengagement and self-efficacy primarily when individuals possess sufficient internal resources to process, regulate, and interpret their disengagement experiences. Rather than acting as a straightforward protective factor, hope, resilience, and optimism may shape how disengagement is appraised and how strongly it is associated with perceived self-efficacy. Thus, the results extend prior perspectives in psychology by suggesting that psychological resources may not attenuate the harmful associations linked to disengagement but instead may shape the conditions under which these associations become more pronounced.

The findings further demonstrate that emotional competence is a context-sensitive process that integrates behavioural coping patterns with internal resource states. Accordingly, they suggest a more nuanced conceptualization of coping-related constructs: psychological resources may amplify the significance of maladaptive strategies by enabling deeper emotional awareness and cognitive appraisal, which subsequently affect self-efficacy.

From an applied perspective, the model highlights emotional competence as a strategic target for interventions aimed at enhancing self-efficacy in academic and organizational settings. Programs that focus exclusively on increasing hope, resilience, or optimism without addressing maladaptive coping patterns may be insufficient. In contrast, integrated interventions that simultaneously reduce disengagement and strengthen emotional competence are likely to be more effective, including in academic contexts.

This research also presents limitations that should be considered when interpreting the results. First, although the sample of 131 participants was adequate for the analyses conducted, the study was restricted to undergraduate students from a single university, which limits the generalizability of the findings to other contexts. Second, the cross-sectional design constrains causal inference, or temporal ordering, and the reliance on self-report measures represents an additional limitation. Moreover, the behavioural disengagement measure captured a general coping tendency rather than a state-specific response to a particular academic stressor. Coping in STEM environments may vary across contexts and time points, and future research would therefore benefit from state-based, course-specific, or repeated-measures approaches capable of capturing the dynamic nature of disengagement under high-pressure academic conditions. Reverse or reciprocal associations among behavioral disengagement, emotional competence, and self-efficacy are also plausible and should be examined in future longitudinal research.

Nevertheless, the findings support the relevance of emotional competence as a factor associated with self-efficacy and indicate that the relationship between disengagement and self-efficacy is conditional on psychological resources such as hope, resilience, and optimism. In conclusion, by integrating coping, emotional, and positive psychological processes into a single framework, this study offers a differentiated understanding of how adaptive beliefs may be linked to disengagement experiences in demanding academic contexts.

## Data Availability

The datasets presented in this study can be found in online repositories. The names of the repository/repositories and accession number(s) can be found at: https://osf.io/h7p65/.
